# Neonatal severe hyperparathyroidism due to a homozygous mutation of calcium-sensing receptor; a challenging case

**DOI:** 10.4038/cmj.v64i4.8988

**Published:** 2019-12-31

**Authors:** Raihana Hashim, Michael A Levine, Kala Somasundarum, Ananda Lamahewage, Navoda Atapattu

**Affiliations:** 1 Senior Registrar in Paediatric Endocrinology, Lady Ridgeway Hospital, Colombo, Sri Lanka,; 2 Professor of Paediatrics and Medicine, University of Pennsylvania Perelman School of Medicine and Division of Endocrinology and Diabetes, Children’s Hospital of Philadelphia,; 3 Consultant Paediatrician, Lady Ridgeway Hospital, Colombo, Sri Lanka,; 4 Consultant Paediatric Endocrinologist, Lady Ridgeway Hospital, Colombo, Sri Lanka.

**Keywords:** neonatal severe hyperparathyroidism (NSHPT), homozygous calcium sensing receptor (CaSR) mutations, cinacalcet, parathyroid auto-transplantation

## Background

The key sensor of extracellular ionised calcium (Ca) is the calcium-sensing receptor (CaSR); the key physiological actions being regulation of parathyroid hormone (PTH) release from the parathyroid glands and calcium reabsorption from the renal tubules [1].

Inactivating mutations of the CaSR gene reduce the receptor sensitivity to Ca resulting in increased PTH secretion, reduced renal calcium excretion, and hypercalcaemia. Bi-allelic CaSR mutations typically lead to neonatal severe hyperparathyroidism (NSHPT) [2].

The standard therapy for NSHPT is total parathyroidectomy [3]. Recent preliminary reports suggest improved outcomes with contemporaneous parathyroid auto-transplantation [4]. Here we describe a Sri Lankan infant with NHSPT due to a homozygous CaSR mutation who was successfully treated at 3 months of age by total parathyroidectomy and contemporaneous parathyroid auto-transplantation.

### Case presentation and results

A 10-day old neonate presented with low-grade fever, poor feeding, reduced activity and constipation of 3-day duration. There were no associated seizures. He was born at term with a birth weight of 3.6kg to second-degree consanguineous parents and had no family history of hypercalcaemia, bone disease, or renal stones. On admission, he was lethargic and hypotonic, and had moderate dehydration with a 30% weight loss. No subcutaneous fat necrosis was felt.

Investigations revealed severe hypercalcaemia, low serum phosphorus, normal alkaline phosphatase (ALP), high serum intact parathormone (PTH) with a sufficient Vitamin-D level (Table 1). Radiographs of the hand and chest revealed generalised osteopenia and periosteal erosions without evidence of fractures.

The immediate management involved hyper-hydration and intravenous furosemide, followed by IV pamidronate administration, resulting in an improvement in hypercalcaemia, the infant’s symptoms and weight. He was discharged on oral furosemide.

At 6 weeks of age, furosemide was stopped as hypercalcaemia worsened and a trial of cinacalcet was initiated (1.5 mg/kg/d). Increasing cinacalcet doses (maximum dose 11mg/kg/day) failed to show a sustained response, therefore at 3 months of age and with a body weight of only 3.6 kg, surgery was performed. The PTH level had increased to 1744 pg/ml. During surgery, the paediatric surgeon identified four enlarged parathyroid glands and 24 hours after total parathyroidectomy serum PTH was undetectable. Half of a parathyroid gland was re-implanted under the left biceps muscle.

Calcitriol which was started postoperatively was gradually reduced and totally eliminated at 16-weeks post-surgery. Serum calcium and PTH (16 pg/mL) were normal, but serum phosphorus was elevated, suggesting mild parathyroid insufficiency. Serum calcium remained normal till 18-months postoperatively when hypocalcaemia with a low PTH level was detected, and calcitriol was re-started.

After surgery the child showed impressive catch up growth, and at 18-months his neurodevelopment was age-appropriate. There was no biochemical or radiological evidence in renal ultrasound of hypercalciuria secondary to calcium therapy.

After parathyroidectomy the diagnosis of NSHPT was confirmed by genetic testing that revealed a previously reported (3) homozygous nonsense mutation in CaSR: NM_000388.3: c.679 C>T: p. R227X which predicts a truncated protein that would be non-responsive to calcimimetic therapy. Both parents were heterozygous carriers. Family screening of 1st degree relatives revealed normocalcemia and normocalciuria (Table 1).

## Discussion

NSHPT is a rare genetic disorder which presents in the first few weeks of life with poor feeding, lethargy, hypotonia, respiratory distress and failure to thrive, all of which are non-specific symptoms and signs of common neonatal problems [2]. Failure to recognise the problem early and initiate appropriate treatment promptly is associated with poor neurodevelopmental outcomes and a mortality rate of greater than 50% [3].

In the absence of widely-available rapid molecular testing, a diagnosis relied on identifying the typical clinical presentation and biochemistry. A typical presentation and severe hypercalcaemia in an infant born to consanguineous parents were highly suggestive of NHSPT, and further testing which revealed high PTH levels and typical radiological features was highly suggestive in our patient.

The differential diagnosis of neonatal hypercalcaemia includes idiopathic infantile hypercalcaemia, William syndrome, subcutaneous fat necrosis, vitamin D intoxication and hypophosphatasia; all of which are associated with low PTH levels.

Cinacalcet is an allosteric activator of the CaSR, increasing its sensitivity to extracellular calcium leading to a decrease in PTH output. Homozygous inactivating mutations of CaSR are known to be non-responders to calcimimetics [5], as seen in our patient.

In patients whom cinacalcet was successful, a significant calcium reduction was seen within a couple of days. A dramatic response in the PTH and calcium levels were seen 2 days after cinacalcet (0.4mg/kg/day) [6]. Therefore, in the absence of rapidly-available genetic testing, a lack of response to a trial of cinacalcet should suggest homozygous mutations.

In conclusion, this case highlights the importance of recognising a rare disorder associated with non-specific manifestations and initiating timely and appropriate specialized management in order to achieve desirable outcomes. Furthermore, in the absence of rapid molecular evaluation, a short trial of cinacalcet is desirable. Failure to show a dramatic reduction in calcium levels within a few days of initiating cinacalcet should indicate the need for total parathyroidectomy, which is life-saving.

## Figures and Tables

**Table 1.** Illustrates the biochemistry of the patient and his 1^st^ degree relatives

Lab test	Patient	Father	Mother	Sister 1	Sister 2	Sister 3
Serum albumin-corrected calcium (mmol/L)	7.43	2.34	2.40	2.48	2.55	2.32
Reference range (2.20–2.70)						
Serum Phosphate (mmol/L)	0.75	0.90	1.18	1.23	1.46	1.51
Reference ranges						
< 2 years (1.45–2.16)						
2–12 years (1.45–1.78)						
>12 years (0.87–1.45)						
Alkaline phosphatase (U/L)	246	-	-	-	-	-
Reference ranges:						
Children (80–480)						
Adults (100–360)						
Serum intact parathormone (pg/ml)	403	-	-	-	-	-
Reference range: 14–72						
25-hydroxy vitamin D (nmol/L)	50.2	-	-	-	-	-
Reference range:						
Sufficient level >50 nmol/L						
Urine calcium/creatinine ratio	-	0.29	0.21	0.15	0.02	0.38
Hypocalciuria < 0.01						
Hypercalciruia > 0.2						
